# Design of Covert Communication Waveform Based on Phase Randomization

**DOI:** 10.3390/e27050520

**Published:** 2025-05-13

**Authors:** Wenjie Zhou, Zhenyong Wang, Jun Shi, Qing Guo

**Affiliations:** School of Electronic and Information Engineering, Harbin Institute of Technology, Harbin 150006, China; 22b305003@stu.hit.edu.cn (W.Z.).com; junshi@hit.edu.cn (J.S.); qguo@hit.edu.cn (Q.G.)

**Keywords:** LPI, NRPCS, LPD, covert communication, security

## Abstract

Covert wireless communication is designed to securely transmit hidden information between two devices. Its primary objective is to conceal the existence of transmitted data, rendering communication signals difficult for unauthorized parties to detect, intercept, or decipher during transmission. In this paper, we propose a Noise-like Multi-Carrier Random Phase Communication System (NRPCS) to enhance covert wireless communication by significantly complicating the detection and interception of transmitted signals. The proposed system utilizes bipolar modulation and Cyclic Code Shift Keying (CCSK) modulation, complemented by a random sequence generation mechanism, to increase the randomness and complexity of the transmitted signals. A mathematical model of the NRPCS waveform is formulated, and detailed analyses of the system’s time-domain basis functions, correlation properties, and power spectral characteristics are conducted to substantiate its noise-like behavior. Simulation results indicate that, compared to traditional fixed-frequency transmission methods, NRPCS substantially improves both the Low Probability of Detection (LPD) and the Low Probability of Interception (LPI). Further research results demonstrate that unauthorized eavesdroppers are unable to effectively demodulate signals without knowledge of the employed modulation scheme, thus significantly enhancing the overall security of communication.

## 1. Introduction

With the rapid advancement of modern communication technologies, wireless communication has become the predominant mode of communication in both civilian and military domains. However, due to the inherent openness of the wireless communication medium, transmitted signals are vulnerable to interception by unauthorized third parties. Consequently, as wireless communication technology becomes increasingly prevalent, it faces escalating security challenges. The primary objective of covert communication is to conceal the existence of transmitted information, making communication signals difficult to detect, intercept, or decipher by unauthorized entities during transmission. Traditionally, encryption methods based on cryptography are employed to secure transmitted information, ensuring that intercepted signals cannot be accurately interpreted by third-party eavesdroppers. Nonetheless, with the continuous enhancement of computational capabilities, encryption technologies relying solely on computational complexity have begun to reveal their limitations. Therefore, covert communication, characterized by its ability to obscure the presence of information transmission itself, has emerged as a significant area of research in information security.

Covert communication, also known as low probability of detection (LPD) communication [1], aims primarily at preventing malicious eavesdroppers from detecting communication signals. Even if intercepted, such signals should remain indistinguishable from typical noise or background signals, thereby obscuring their recognition as legitimate data transmission. Spread spectrum technology is frequently employed in covert communications, transmitting signals over a wide frequency band to minimize detection probability [2]. Recently, however, with the increasing sophistication of security threats, traditional spread spectrum methods have become insufficient, prompting the development of noise-based communication systems [3]. Unlike conventional coding and physical-layer security techniques, noise-based communication emphasizes concealing the existence of information transmission [4], thereby safeguarding transmitted content from discovery. Various approaches have been proposed in the recent literature to achieve effective covert communication.

Reference [5] addresses spiking noise found in certain communication channels by proposing a wireless covert communication method that simulates spiking noise through embedding information within pulse characteristic parameters. Gaussian noise represents the most prevalent noise type in practical channels. Reference [6] suggests embedding information into the correlation coefficients of consecutive Gaussian sequences, allowing receivers to demodulate signals based on these coefficients. Another approach involves modulating covert information into interference signals, simultaneously delivering the concealed message and disrupting adversary communications. Although implementation specifics vary, these methods leverage signal or noise characteristics to achieve covert transmission by subtly altering signal properties, complicating detection efforts.

Considering the unpredictable nature of wireless channels and the evolving capabilities of adversaries equipped with advanced anti-encryption tools and powerful computational resources, the disclosure of encryption keys may severely compromise traditional encryption methods, thus necessitating enhanced security mechanisms [7]. Consequently, covert communication employing noise-like waveforms not only exhibits strong concealment and interception resistance but also supports minimal communications under critical conditions. This approach offers robust adaptability, providing substantial protection for various security-sensitive applications.

Reference [8] introduces a method that superimposes hidden signals onto standard signal constellation diagrams through quasi-modulation. Similarly, Reference [9] overlays covert information onto known Orthogonal Frequency Division Multiplexing (OFDM) data. Reference [10] presents orthogonal chaotic shift keying to modulate covert signals, subsequently superimposed onto regular communication signals, effectively addressing low covert signal power issues identified in Reference [11] and enhancing covert transmission performance.

Signal processing technologies can further enhance covert communications. Reference [12] employs Weighted Fractional Fourier Transform (WFRFT), exploiting constellation rotation and contraction characteristics to improve concealment. Reference [13] proposes a multi-parameter weighted fractional Fourier transform cryptographic modulation method, significantly enhancing security compared to single-parameter WFRFT. Additionally, Reference [14] introduces a two-layer satellite chaotic phase modulation scheme utilizing two multi-parameter WFRFT (MP-WFRFT) operations. Reference [15] conducts a detailed analysis of MP-WFRFT parameters’ influence on signal constellation patterns and proposes a constellation pre-coding mechanism employing MP-WFRFT to produce diverse constellation patterns, thereby confusing eavesdroppers.

This paper proposes a covert communication method based on a multi-carrier noise-like random phase communication system. Utilizing time-domain basis functions generated by chaotic pseudorandom sequences, the system transforms signals into noise-like signals through symbolic spread spectrum techniques. The transmitter subsequently transmits these noise-like signals to the receiver for demodulation. After processing, the covert signals exhibit low energy and Gaussian characteristics, allowing effective blending with background noise and enhancing their interception resistance. Simultaneously, synchronization and channel estimation within the communication system can be effectively achieved using publicly transmitted signals.

The paper thoroughly describes the implementation process of the proposed method and details the structural components involved. It theoretically derives the power spectrum and Peak-to-Average Power Ratio (PAPR), analyzes the correlation characteristics of noise-like signals, evaluates the bit error rate (BER) performance of covert signals, and verifies their concealment features. Simulation experiments are conducted to comprehensively assess the performance of the proposed system.

Compared with traditional spread spectrum communication methods, the innovative aspects of the multi-carrier noise-like random phase communication system include advanced multi-carrier signal processing, effective generation of noise-like signals, and improved anti-interference capabilities. Through meticulous design of the noise-like signals, the proposed system demonstrates significant advantages in concealment, interception resistance, spectrum efficiency, and signal quality.

The remainder of this paper is organized as follows: Section 2 presents the system model. Section 3 provides a detailed theoretical analysis of key parameters, including a comparative evaluation of critical metrics such as the bit error rate of covert signals. Finally, conclusions are presented in Section 4.

## 2. System Model

### 2.1. Basic Composition and Mathematical Model of NRPCS

**Figure 1:**
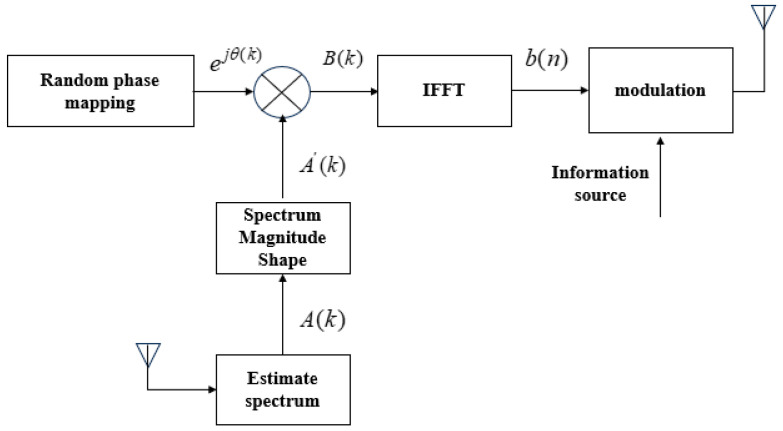
NRPCS sending block diagram.

A system block diagram provides a clear and intuitive understanding of the entire system’s operation. Figure 1 presents the transmitter block diagram of the Noise-like Multi-Carrier Random Phase Communication System (NRPCS). The transmitter primarily comprises modules for amplitude spectrum formation, random phase mapping, time-domain basis function generation via an inverter, data modulation, data memory, radio frequency transmission, and antenna components. Additionally, modules for energy and power regulation as well as storage are included to facilitate efficient signal processing. At the core of the NRPCS is the construction of basic modulation waveforms possessing specialized characteristics. Specifically, waveforms are generated through random phase mapping and amplitude spectrum shaping, subsequently transformed to synthesize time-domain basis functions exhibiting covert characteristics. Data modulation is then achieved by mapping these synthesized time-domain basis functions in various manners, enabling effective information transmission. The NRPCS utilizes random-phase waveforms, which demonstrate prominent noise-like characteristics, significantly enhancing covert communication capabilities and physical-layer security.

As illustrated in Figure 1, the basic implementation process of NRPCS is as follows: the electromagnetic environment is dynamically sampled within a designed system bandwidth, after which spectrum estimation is performed. The resulting estimated amplitude spectrum vector A(k) is then evaluated against a predetermined threshold to identify spectrum segments experiencing interference and those that remain unoccupied, thereby suitable for signal transmission. By employing a targeted thresholding technique, interference is effectively suppressed, producing a “clean” amplitude spectrum vector $A^{\prime }(k)$ that excludes interfered or occupied frequency components. This step is known as amplitude spectrum shaping. The vector $A^{\prime }(k)$ is then multiplied by a complex random phase vector of equal length $e^{j\theta (k)}$, generated by a phase mapper. The result is B(k), which serves the purpose of assigning a random phase to each available frequency point, giving the time-domain communication signal a noise-like characteristic.

**Figure 2:**
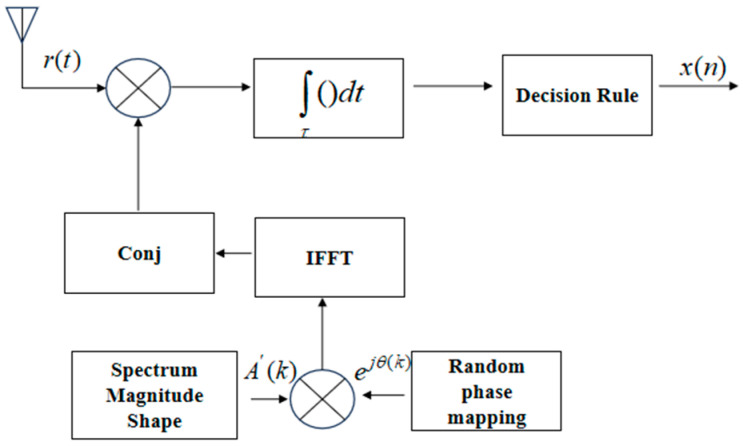
NRPCS receiving block diagram.

The receiver structure of the NRPCS is depicted in Figure 2, representing the inverse operation of the transmitter. The receiving end primarily comprises components such as an antenna, RF receiver, data cache and synchronization modules, a peak finder, and demodulation circuits. To maintain consistency with the fundamental modulation waveform produced by the transmitter, the receiver must similarly execute procedures like amplitude spectrum generation and random phase mapping to reconstruct the time-domain basis functions. Amplitude spectrum generation ensures that the frequency-domain characteristics of the received signals match those of the transmitted modulation signals. Random phase mapping at the receiver aims to compensate for and correct random phase shifts introduced during signal transmission. Phase variations may occur due to environmental noise, multipath propagation effects, or other environmental factors. Thus, accurate phase correction at the receiver is essential for effective demodulation and accurate retrieval of the transmitted data.

Each CCSK sequence is generated through a periodic shift of the base sequence. According to this feature, the receiver employs a cyclic correlation detection method for signal recovery [16]. Specifically, the received signal undergoes a cyclic correlation operation with the locally stored base sequence. By analyzing the positional difference between the resultant correlation peak and the inherent peak of the base sequence, the receiver determines the shift value of the CCSK codeword. Consequently, the corresponding data information encoded in the shifted code is effectively extracted.

### 2.2. Amplitude Spectrum Molding

Amplitude spectrum shaping is a technique used to optimize the signal transmission spectrum. It involves comparing the environmental spectral information matrix with a predefined threshold to determine which frequencies within a specified bandwidth are unavailable and which remain unoccupied and suitable for transmission. This procedure results in a usable spectral matrix, serving as the amplitude spectrum for the corresponding time-domain basis function.

According to the findings presented in Reference [17]. The amplitude spectrum design primarily determines the amplitude of the Fundamental Modulation Waveform (FMW). Specifically, the amplitude spectrum is derived from processing the Power Spectral Density (PSD) estimated from the signal spectrum. The threshold utilized in this context is determined based on the average power across the system bandwidth, effectively suppressing interference spectral components. Currently, two primary methods are employed for spectrum shaping: flat amplitude molding and coded amplitude molding.

In flat amplitude molding, if the amplitude of an interference spectrum exceeds the preset threshold, the amplitude at that frequency point is set to zero; otherwise, it is set to one, resulting in an FMW amplitude spectrum composed exclusively of binary values (0 or 1). Conversely, coded amplitude molding takes channel conditions into account by setting the amplitude spectrum based on the channel transmission function. In this approach, if the interference amplitude surpasses the threshold, the spectral component’s amplitude is set accordingly.

According to the findings presented in Reference [18]. Previous research has applied coded amplitude molding to analyze spectrum shaping performance under multipath fading channels. The findings indicate that, when the number of multipath components varies from 2 to 50, the coded amplitude molding method achieves an improvement in Signal-to-Noise Ratio (SNR) of approximately 1 to 2.75 dB compared to flat amplitude molding. Furthermore, in practical applications, Doppler frequency shifts also represent a significant channel factor that must not be neglected. Therefore, investigating spectrum shaping techniques under Doppler frequency shift conditions remains essential for comprehensive performance assessment.

### 2.3. Noise-like Random Phase Signal Generation Method

This section focuses on the generation method of noise-like random phase signals. Random phase mapping serves three primary purposes in the NRPCS system. First, it imparts noise-like characteristics to the transmitted signals, thereby enhancing the system’s low probability of detection and low probability of interception. Second, the cross-correlation between different random phase sequences is minimal, while the autocorrelation of an individual sequence is highly prominent. This property supports the system’s multiple access capability. Third, the inherent correlation characteristics facilitate efficient modulation and demodulation processes.

**Figure 3:**
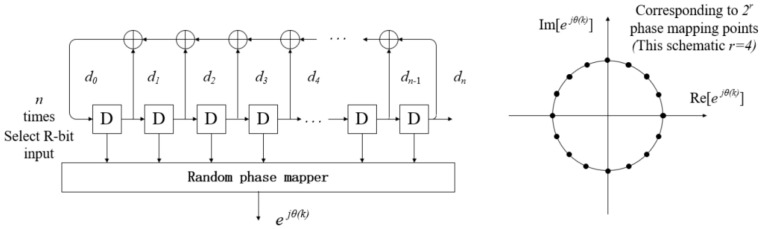
Schematic diagram of random phase mapping.

In random phase communication systems, random phase mapping is often implemented using m-sequences. As shown in Figure 3, these sequences are generated based on the cyclic operation of a Linear Feedback Shift Register (LFSR) [19]. To begin, the number of register stages n must be determined, and a primitive polynomial—one that is irreducible and possesses a maximum period—is selected as the feedback logic coefficient. During initialization, the register is typically set to all zeros, except for the last bit, which is initialized to one to avoid the all-zero deadlock state. In each shift operation, a new bit is produced by performing XOR operations on specific register bits, as dictated by the coefficients of the primitive polynomial. Subsequently, all register states are shifted one position to the right, and the newly generated bit is inserted into the leftmost register position.

During the phase mapping process, r registers are selected from the n-stage LFSR to serve as the input to the phase mapper. These r register values can map up to $2^{r}$ random phase points. The selection of the r registers can be any arbitrary combination or a consecutive set of registers. Consecutive selection helps ensure a more uniform distribution of values in the resulting pseudo-random multivariate sequence.

Let the generated random phase sequence be

(1) $$\begin{array}{c} e(k)=\left[\begin{array}{cccccc} e^{j\theta_{0}(k)} & e^{j\theta_{1}(k)} & \cdots & e^{j\theta_{i}(k)} & \cdots & e^{j\theta_{N-1}(k)} \end{array}\right]\\ \theta_{i}(k)=\frac{2\pi n_{i}}{N} \end{array}$$

among i=0, 1, 2, ⋯ , N−1, $n_{i}\in \left[0,\;1,\;2,\;\cdots \;,\;N-1\right]$, $N=2^{r}$.

By altering the primitive polynomial configuration of the Linear Feedback Shift Register (LFSR), multiple distinct pseudo-random polyphase sequences can be generated through random phase mapping. These sequences serve as the basis for constructing different fundamental modulation waveforms. By assigning specific pseudo-random polyphase sequences to individual users and leveraging the quasi-orthogonality among the resulting fundamental modulation waveforms, multi-user access can be effectively achieved in a random phase communication system.

### 2.4. Time Domain Basis Function Generation

Time-Domain Basis Function Generation refers to the process of constructing fundamental time-domain waveforms that act as the foundational elements for signal modulation in communication systems. These basis functions are essential for shaping the characteristics of the transmitted signal. The corresponding frequency-domain basis function matrix is obtained by performing an element-wise multiplication (inner product) between the constructed amplitude spectrum matrix $E^{\prime }(k)$ and the generated random phase matrix e(k). This operation yields a composite frequency-domain representation, which is then transformed into the time domain to produce the desired basis functions with noise-like properties [20].

(2) $$B^{\prime }(k)=E^{\prime }(k)\cdot e(k)$$

Due to the uncertainty in the number of subcarriers available within the usable spectrum, it is necessary to perform amplitude adjustment of the basis function in the frequency domain. This adjustment is crucial to mitigate excessive power fluctuations that may arise from variations in the spectral matrices. By normalizing the amplitude, the system not only reduces circuit complexity but also eliminates the requirement for automatic gain control prior to signal amplification at the transmitter end. Here, the adjustment factor $\lambda =\sqrt{N/N_{1}}$ is introduced, where N is all the frequency points and $N_{1}$ is the available frequency points, thus the frequency domain basis function after amplitude adjustment is

(3) $$B(k)=\lambda B^{\prime }(k)=\sqrt{N/N_{1}}E(k)\cdot e^{j\theta (k)}$$

The Inverse Discrete Fourier Transform (IDFT) is subsequently applied to the frequency-domain basis function to generate the corresponding time-domain basis function sequence. This transformation facilitates the conversion of the designed frequency-domain representation into a time-domain waveform suitable for transmission.

(4) $$b(n)=IDFT[B(k)]=\frac{1}{\sqrt{N\cdot N_{1}}}\sum_{k=0}^{N-1}E(k)e^{j\theta (k)}e^{j\frac{2\pi kn}{N}}$$

The generated time-domain basis function is stored for reuse, provided that the external environment remains unchanged. This storage capability enables efficient reuse of the waveform without repeated computation, and facilitates further modification and dynamic adjustment of the amplitude, power, and phase of the time-domain basis function. Additionally, it supports subsequent modulation processes and enables flexible control of the data transmission rate.

Suppose that the time domain basis functions generated by two different random phases are $b_{1}(n)$ and $b_{2}(n)$ respectively, and the cross-correlation function can be obtained by the definition of the cross-correlation function

(5) $$R_{12}(m)=E[b_{1}(n)b_{2}^{*}(n)]=\frac{1}{N^{2}}\sum_{k=0}^{N-1}{\left|E^{\prime }(k)\right|}^{2}e^{j[\theta_{1}(k)-\theta_{2}(k)]}e^{j\frac{2\pi km}{N}}$$

where m is the relative time shift between two different time-domain basis function.

Based on the definition of the autocorrelation function, the autocorrelation of the time-domain basis function can be expressed as follows:

(6) $$R(m)=E[b(n)b(n+m)]=\frac{1}{N^{2}}\sum_{k=0}^{N-1}{\left|E^{\prime }(k)\right|}^{2}e^{j\frac{2\pi km}{N}}$$

When m=0, the maximum value of the autocorrelation function i.

(7) $$R_{\max}(m)=R(0)=\frac{1}{N^{2}}\sum_{k=0}^{N-1}{\left|E^{\prime }(k)\right|}^{2}$$

Based on the multistage shift register, two distinct pairs of m-sequences are generated using two different primitive polynomials and initial states, as determined by the previously described formula. From each interval, 3 consecutive bits are extracted and mapped to one of 8 distinct random phase mapping points. A total of 128 random phases are selected in this manner to construct the random phase matrix.

(8) $$\begin{array}{c} f_{0}(x)=x^{6}+x+1\\ a_{0}=[\begin{array}{cccccc} 0 & 0 & 1 & 0 & 0 & 1 \end{array}] \end{array}$$

(9) $$\begin{array}{c} f_{1}(x)=x^{6}+x^{5}+1\\ b_{0}=[\begin{array}{cccccc} 1 & 0 & 0 & 0 & 0 & 1 \end{array}] \end{array}$$

The above formula outlines a pseudorandom sequence generation process based on a primitive polynomial and a shift register, where f(x) specifies the register bits involved in determining the output, and $a_{0}$ represents the initial state of the register. The sequence is generated by cyclically shifting the register and extracting values at defined intervals, which are subsequently mapped to corresponding random phase values.

### 2.5. Modulation and Demodulation of NRPCS

This section focuses on transferring the signal from the time domain to the frequency domain to enable efficient data transmission and processing.

While traditional communication systems typically employ carrier modulation, the NRPCS achieves modulation through waveform modulation, specifically by transforming the generated time-domain basis functions. The modulation techniques used in noise-like communication systems primarily include bipolar modulation and orthogonal modulation. An enhanced version of Cyclic Code Shift Keying (CCSK) modulation, which builds upon these two methods, is adopted to improve system performance.

CCSK modulation transmits data by cyclically shifting a basis function waveform that possesses favorable periodic autocorrelation properties. Let the time-domain basis function sequence of length N be defined as follows:

(10) $$s_{0}=\left[\begin{array}{cccccc} b_{0} & b_{1} & \cdots & b_{k} & \cdots & b_{N-1} \end{array}\right]$$

The corresponding cyclic shift sequence can be expressed as:

(11) $$\begin{array}{c} s_{0}=\left[\begin{array}{cccccc} b_{0} & b_{1} & \cdots & b_{k} & \cdots & b_{N-1} \end{array}\right]\\ s_{1}=\left[\begin{array}{cccccc} b_{1} & b_{2} & \cdots & b_{k} & \cdots & b_{0} \end{array}\right]\\ s_{2}=\left[\begin{array}{cccccc} b_{2} & b_{3} & \cdots & b_{k} & \cdots & b_{1} \end{array}\right]\\ \cdots \\ s_{N-1}=\left[\begin{array}{cccccc} b_{N-1} & b_{0} & \cdots & b_{k} & \cdots & b_{N-2} \end{array}\right] \end{array}$$

Each sequence can represent a data information, N is generally a multiple of 2, so N elements can represent at most $\log_{2}N$ bits of data information. For example, if the sequence length N=32 and the modulation order M is 32, the CCSK modulation mapping is shown in Table 1.

Multi-base CCSK modulation imposes stringent requirements on synchronization. In practical applications, correlation operations are typically employed during demodulation to detect correlation peaks. However, if there is any delay in the occurrence of these peaks, their positions may be misidentified, potentially leading to demodulation errors.

Therefore, the sequence length N is generally set to be greater than the modulation order M, so that the number of cyclic shift elements is often greater than 1. As shown in Table 2 is the CCSK modulation mapping mode with sequence length N=128 and modulation order M=32. Cyclic shift every 4 code elements represent a data to be transferred.

**Figure 4:**
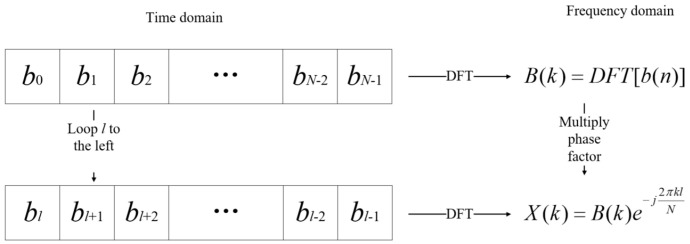
Correspondence between CCSK modulation in time domain and frequency domain.

A time-domain cyclic shift is mathematically equivalent to multiplication by a phase factor in the frequency domain. As illustrated in Figure 4, CCSK modulation can also be implemented through frequency-domain multiplication.

CCSK modulation can be interpreted as a specialized form of spread spectrum communication that employs a coding-like approach to simultaneously achieve spectrum spreading and information transmission. Compared to binary Direct Sequence Spread Spectrum (DSSS), CCSK can achieve a data transmission rate that is times higher, where M is the number of CCSK elements, given the same channel bandwidth and spreading code length.

The selection of the CCSK basis function is critical. To facilitate demodulation at the receiver, it is generally required that the main peak of the periodic autocorrelation function of the basis function is high, while the peak of the periodic cross-correlation function is low. These properties are essential for accurate demodulation and to support multiple access in the system.

Correlation-based demodulation is typically employed in CCSK systems. Two common implementation approaches include matched filtering and frequency-domain demodulation. The frequency-domain method transforms the time-domain convolution operation into a frequency-domain multiplication, offering significant advantages in terms of hardware efficiency and implementation complexity.

The CCSK correlation demodulation method is primarily based on the fundamental principle of correlation detection. For an m-order CCSK system, both the receiver and transmitter generate the time-domain basis function and its cyclically shifted versions. Each of these is then multiplied and integrated with the received signal, followed by sampling at time t=T.

Let the transmitted signal be denoted as s(t). Assuming the channel is an additive white Gaussian noise (AWGN) channel, the received signal can be expressed as

(12) r(t)=s(t)+n(t)

A set of received signals are first multiplied by the conjugate of the basis function and then integrated

(13) $$\begin{array}{ll} f(t) & =\int_{0}^{T}r_{j}(t)s^{*}(t)dt=\int_{0}^{T}\left[s_{j}(t)+n(t)\right]s_{i}^{*}(t)dt\\ & =\int_{0}^{T}s_{j}(t)s_{i}^{*}(t)dt+\int_{0}^{T}n(t)s_{i}^{*}(t)dt \end{array}$$

where n(t) is Gaussian white noise, $s_{i}^{*}(t)$ is the conjugate of the basis function, the two are independent of each other, the integral result is approximately 0, the above equation can be further simplified to

(14) $$f(t)=\int_{0}^{T}s_{j}(t)s_{i}^{*}(t)dt$$

When the received signal has the same number of shifts as the multiplied basis function (i=j), the output is a constant; the output is approximately 0.

(15) $$\begin{array}{cc} f(t) & =\int_{0}^{T}s_{j}(t)s_{i}^{*}(t)dt\\ & =\left\{\begin{array}{ll} K & i=j\\ 0 & i\neq j \end{array}\right. \end{array}$$

Sampling is conducted at time t=T. If a peak value is detected at this sampling point, it indicates that data corresponding to this specific path have been transmitted, thereby completing the demodulation process.

The correlation demodulation method for multi-base CCSK is consistent with demodulation techniques used in most modulation schemes. However, it presents certain challenges, notably the requirement for the receiver to reconstruct multiple fundamental modulation waveforms and the necessity for multiple multipliers and integrators to perform correlation operations. These factors result in increased structural complexity and hardware implementation difficulties. Consequently, practical implementations frequently adopt alternative demodulation techniques, such as the matched filter method derived from correlation demodulation and the frequency-domain demodulation method.

The matched filter approach effectively achieves CCSK demodulation by designing an appropriate matched filter at the receiver. The transmitted data are determined by evaluating the relative position of the output correlation peaks generated by the matched filter. Utilizing this approach, the demodulation structure transitions from employing multiple correlators to a single matched filter, significantly simplifying the hardware architecture. Additionally, the availability of FIR Intellectual Property (IP) cores in FPGA implementations further facilitates and simplifies hardware realization.

If the matched filter function is denoted by h(t), the duration of one code element is T, and the basis function is s(t), then the matched filter function can be expressed as follows

(16) $$h(t)=ks_{0}^{*}(T-t)$$

The received signal r(t) is subsequently processed by the matched filter h(t), which is equivalent to performing a convolution operation between the signal and the filter, resulting in the filtered output.

(17) $$\begin{array}{ll} f(t) & =[s(t)+n(t)]*ks_{0}^{*}(T-t)\\ & =ks(t)*s_{0}^{*}(T-t)+kn(t)*s_{0}^{*}(T-t) \end{array}$$

Similar to correlation demodulation, $n(t)*s_{0}^{*}(T-t)$ is approximately 0, so a set of matched filter output signals can be approximated as

(18) $$f_{m}(t)=ks_{m}(t)*s_{0}^{*}(T-t)$$

The received signals represent cyclically shifted versions of the transmitted signal $s_{0}(t)$. Consequently, applying a matched filter to these signals generates correlation peaks. The demodulation process is completed by identifying the locations of these peaks, as each peak corresponds directly to the transmitted data.

The frequency-domain demodulation method operates under the same fundamental principle as the previously described approaches, but differs in that it transforms the signal processing from the time domain into the frequency domain. Specifically, the cyclic correlation operation between two discrete-time signals can be implemented efficiently by frequency-domain multiplication.

(19) $$\begin{array}{cc} f(n) & =r(n)⊙s^{*}(n)=\sum_{n=0}^{N-1}a(n)s^{*}{\left(n+m\right)}_{N}\\ & =r(n)*s^{*}{\left(-n\right)}_{N}=r(n)*s^{*}(N-n) \end{array}$$

It is evident that correlation and convolution operations can be considered equivalent under certain conditions. By applying the Discrete Fourier Transform (DFT) to both sides of the equation above, we obtain the following expression:

(20) $$F(k)=R(k)\cdot S^{*}(k)$$

Therefore, the frequency-domain demodulation method significantly simplifies the complexity of the system by converting intricate correlation and convolution operations into simpler multiplication operations. Specifically, the received signal is initially transformed into the frequency domain using a Fast Fourier Transform (FFT). Subsequently, the conjugate of the base sequence is also converted to the frequency domain through FFT, after which it is multiplied with the frequency-domain representation of the received signal. The resulting product is then transformed back into the time domain using the Inverse Fast Fourier Transform (IFFT) to identify the correlation peak. Finally, the transmitted data can be recovered by comparing the detected peak position with a predefined reference position.

## 3. Results

### 3.1. Time Domain Basis Function Performance Analysis

#### 3.1.1. Power Spectrum and Amplitude Spectrum Analysis

In this section, the power spectrum of the generated random-phase time-domain basis function is analyzed using the direct method. Specifically, the procedure involves performing a Fast Fourier Transform (FFT) on the basis function, squaring the magnitude of the resulting FFT coefficients, and then normalizing by dividing by the number of points N. This yields the power spectrum of the random-phase basis function.

(21) $$PSD=\frac{|FFT[b(n)]{|}^{2}}{N}$$

**Figure 5:**
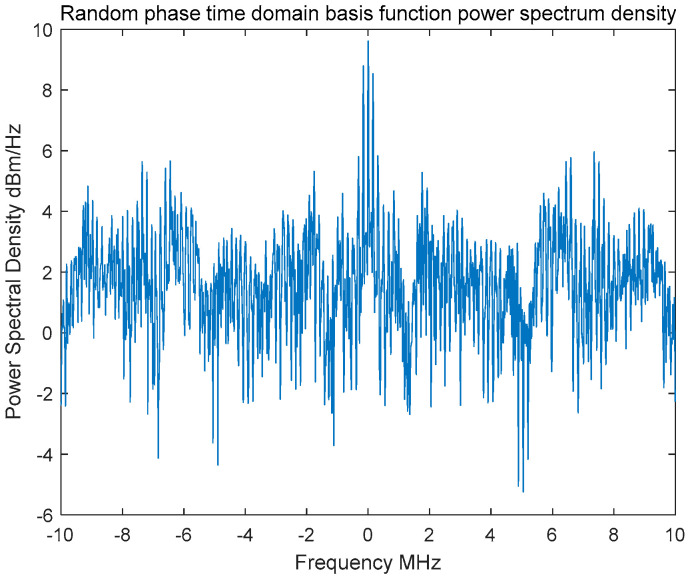
Random phase basis function power spectrum.

In order to thoroughly analyze the characteristics of the fundamental modulation waveform, a sufficiently long random-phase communication signal was generated by employing the basic modulation waveform and its cyclically shifted sequences. Subsequently, the Power Spectral Density (PSD) of the generated signal was estimated using the Welch averaged periodogram method, as illustrated in Figure 5. The Welch method is an advanced power spectrum estimation technique that integrates segment averaging and windowing, effectively balancing spectral resolution with estimation variance, thereby providing an enhanced representation of the power spectral characteristics of the signal.

Due to the utilization of random-phase basis functions for constructing the time-domain signal, its spectrum exhibits irregular fluctuations along the frequency axis, indicating the absence of fixed periodic components. Analysis of the resulting power spectrum curves reveals that the transmitted signal in the random-phase communication system demonstrates a nearly uniform power spectral distribution within its core bandwidth. Specifically, the power spectral density curve appears relatively flat, exhibiting spectral characteristics similar to those of Gaussian white noise. From a power spectrum detection standpoint, this flat spectral characteristic results in a low probability of detection, while simultaneously providing excellent spectral concealment and robust anti-interference performance.

**Figure 6:**
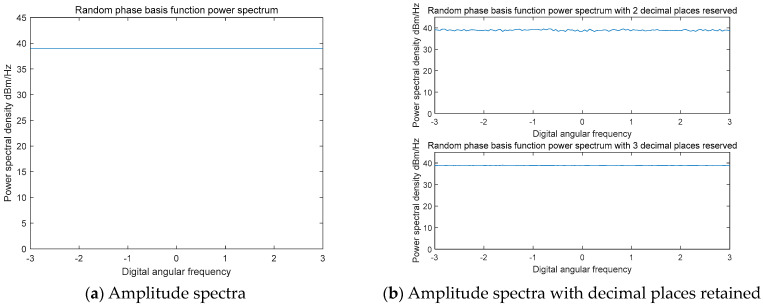
Time domain basis function amplitude spectrum.

By computing the amplitude spectra of the generated time-domain basis function and of the basis function after preserving a certain number of significant digits, the amplitude spectrum of the random-phase fundamental modulation waveform can be derived, as illustrated in Figure 6. The resulting amplitude spectrum of the random-phase modulation waveform remains relatively flat. Furthermore, the amplitude spectrum of the random-phase basis function maintains this flat profile even after truncating to a certain precision level. This observation confirms that the introduction of random phase modulation has minimal impact on the amplitude spectrum of the random-phase basis function.

In the waveform generation process of noise-like random-phase communication systems, the random phases at each frequency point are mutually independent. Consequently, the generated time-domain signals exhibit Gaussian-like distributions according to the central limit theorem. This results in numerous spikes of varying magnitudes within the time-domain waveforms. Various metrics have been proposed in the literature to characterize these spikes, among which the Peak-to-Average Power Ratio (PAPR) is the most widely adopted. PAPR is defined as the ratio of the signal’s maximum instantaneous power to its average power. Mathematically, the PAPR for a noise-like random-phase signal can be expressed as follows:

(22) $$PAPR\left[s_{NRPCS,i}\right]=\frac{\max_{n\in [1,N]}{\left|s_{NRPCS,i}(n)\right|}^{2}}{E\left\{{\left|s_{NRPCS,i}(n)\right|}^{2}\right\}}$$

where E{·} takes an expectation from the traversal signal. In NRPCS, not all frequencies within the system bandwidth are available, but all available frequencies will equally share the energy of the entire signal. Therefore, the peak power, average power of NRPCS transmitted signal and the theoretical upper limit of PAPR can be expressed as:

(23) $$\max_{m\in [1,N]}{\left|s_{NRPCS,i}(n)\right|}^{2}=\max_{n\in [1,N]}{\left|\frac{1}{\sqrt{NN_{1}}}\sum_{k=0}^{N-1}A(k)e^{j\theta }\right|}^{2}\leq \left({\left.\frac{1}{\sqrt{NN_{1}}}\sum_{k=0}^{N-1}\max|A(k)|\right|}^{2}=\frac{A^{2}N_{1}}{N}\right.$$

(24) $$E\left\{{\left|s_{NRPCS,i}(n)\right|}^{2}\right\}=E{\left|\frac{1}{\sqrt{NN_{1}}}\sum_{k=0}^{N-1}A(k)e^{j\theta }\right|}^{2}=\frac{1}{NN_{1}}\sum_{k=0}^{N-1}\left\{E|A(k){|}^{2}\right\}=\frac{A^{2}}{N}$$

(25) $$PAPR\left[s_{NRPCS,i}\right]\leq N_{1}$$

where $e^{j\theta }=e^{j\theta_{k}}e^{-j2\pi m_{l}k/M}e^{j2\pi kn/N}$ and A=1 are the values of the spectral amplitude matrix. It is evident that the Peak-to-Average Power Ratio (PAPR) of the NRPCS system varies not only with changes in the pseudo-random phase but also with variations in the spectral amplitude matrix”.

The Peak-to-Average Power Ratio (PAPR) of the signal is evaluated using the Complementary Cumulative Distribution Function (CCDF), defined as the probability that the system’s PAPR exceeds a specified threshold PAPR0. For symbols in the NRPCS system, the expectation is 0, and assuming the variance of both the real and imaginary parts is $\sigma^{2}$, the instantaneous power follows the $\chi^{2}$ distribution with the expectation of $2\sigma^{2}$.

(26) $$f(z)=\frac{1}{2\sigma^{2}}e^{-\frac{z}{2\sigma^{2}}}$$

For a specific threshold ξ, the peak power of the signal and the peak-to-average ratio of the system can be ex-pressed in the form of CCDF

(27) $$\begin{array}{c} \mathrm{Pr}\left(\max_{n\in [1,N]}{\left|s_{NRPCS,i}(n)\right|}^{2}\leq \xi \right)={\left(1-e^{-\frac{\xi }{2\sigma^{2}}}\right)}^{N_{1}}\\ \mathrm{Pr}\left(PAPR\left[s_{NRPCS,i}\right]>PAPR0\right)=1-{\left(1-e^{-PAPR0}\right)}^{N_{1}} \end{array}$$

**Figure 7:**
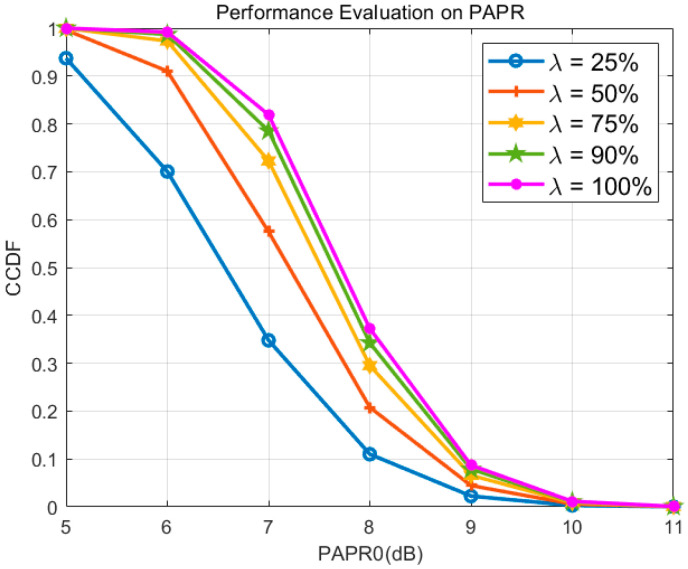
Performance Evaluation on PAPR.

By simulating the aforementioned Peak-to-Average Power Ratio (PAPR) characteristics, as illustrated in Figure 7, it can be observed that a distinctive property of the noise-like random-phase communication system, in contrast to conventional communication systems such as Orthogonal Frequency Division Multiplexing (OFDM), is that the PAPR of the transmitted signal varies with changes in both the pseudo-random phase and the spectral sensing matrix employed by the system. Furthermore, since modulation symbols are generated by cyclic shifts within the system, the PAPR remains consistent across modulation symbols. Consequently, the system’s PAPR remains unaffected by variations in the modulation data.

**Figure 8:**
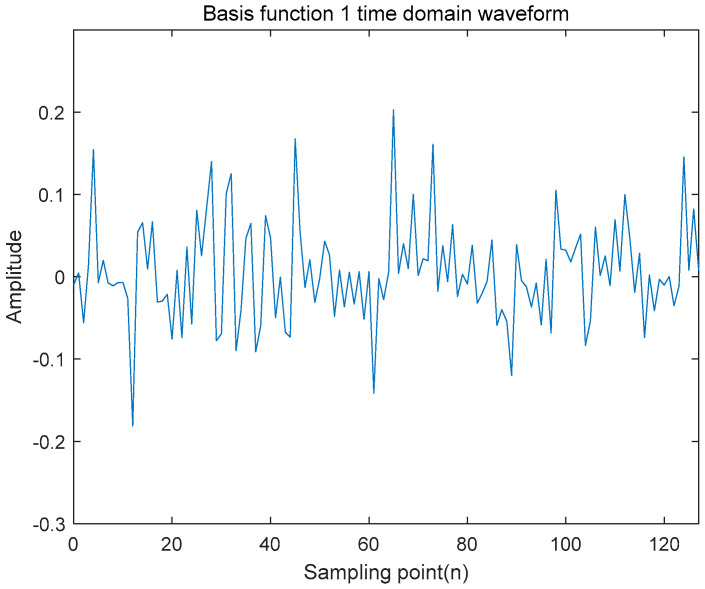
Time domain waveform diagram of basis function.

The simulation parameters are set as N=256, $N_{NRPCS}=8000$. Figure 8 shows PAPR suppression effect of the proposed method under different available spectrum proportions $\lambda =N_{1}/N$. According to Equation (25), the theoretical upper bound of the system’s Peak-to-Average Power Ratio (PAPR) increases with the number of available frequency points. The maximum theoretical PAPR occurs when the instantaneous phases of all available frequency points are aligned; however, the probability of this event occurring is extremely low. Given that simulations typically involve approximately $10^{5}$ iterations, reaching this theoretical upper limit in practice is highly improbable. Nonetheless, the simulation results depicted in the figure clearly illustrate the general trend: the PAPR of the NRPCS is significantly influenced by the number of available frequency points. Specifically, different numbers of available frequency points correspond to distinct curves, and an increase in the number of available frequency points results in a higher system PAPR.

#### 3.1.2. Correlation Characteristic Analysis

To further examine the noise-like characteristics of the system, the correlation properties of the signals were analyzed. Based on a multistage shift register, two pairs of distinct sequences were generated using two different primitive polynomials and initial states, as defined previously. Subsequently, three bits were sequentially extracted from each interval, corresponding to eight distinct random-phase mapping points, resulting in a random-phase matrix composed of 128 random phases.

The generated random-phase matrix was element-wise multiplied with the amplitude spectrum matrix. Initially, considering an interference-free environment, the spectral awareness matrix was set to unity. Following the element-wise multiplication, the time-domain basis function was obtained by performing an Inverse Fast Fourier Transform (IFFT), resulting in the waveform shown in Figure 8. In this simulation, the sequence length was set to 128 points.

**Figure 9:**
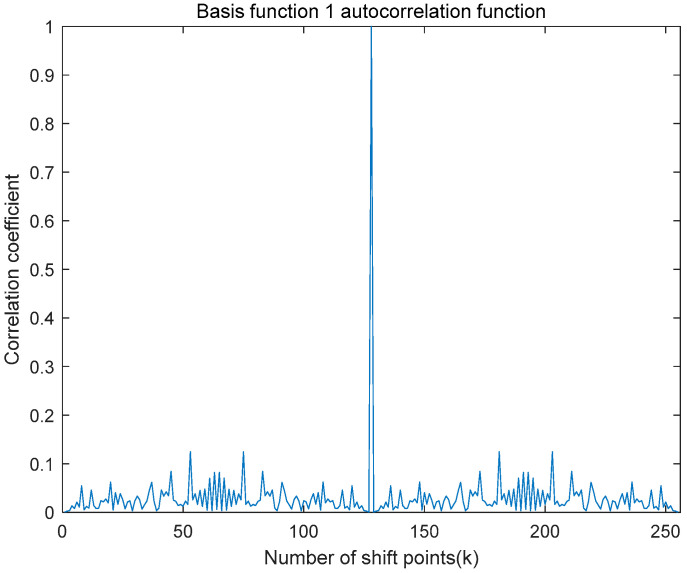
Basis function 1 simulation diagram of autocorrelation function.

**Figure 10:**
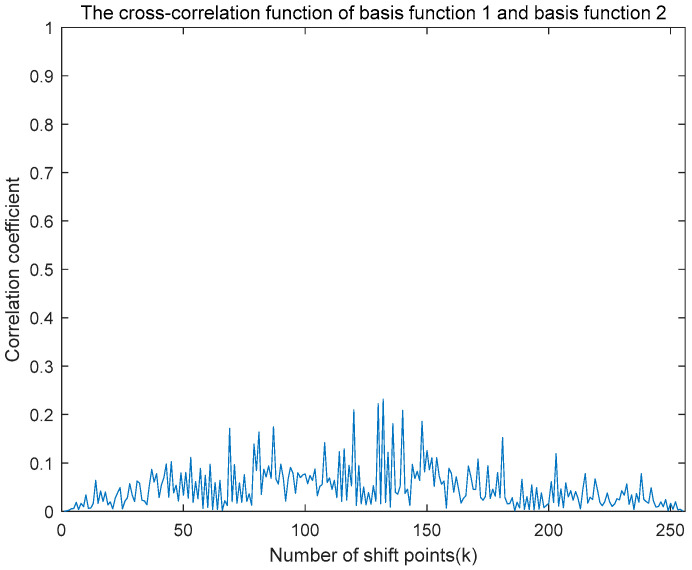
Simulation diagram of cross-correlation function of basis function 1 and basis function 2.

An autocorrelation operation was applied to the first generated set of time-domain basis functions, yielding the autocorrelation function illustrated in Figure 9. Subsequently, cross-correlation operations were performed between the two generated sets of time-domain basis functions, resulting in the cross-correlation function depicted in Figure 10. As clearly demonstrated in these figures, the autocorrelation function displays a distinct and prominent main peak, while the cross-correlation function between the two different basis functions exhibits negligible peaks with amplitudes close to zero. This behavior aligns well with the theoretical expectations.

**Figure 11:**
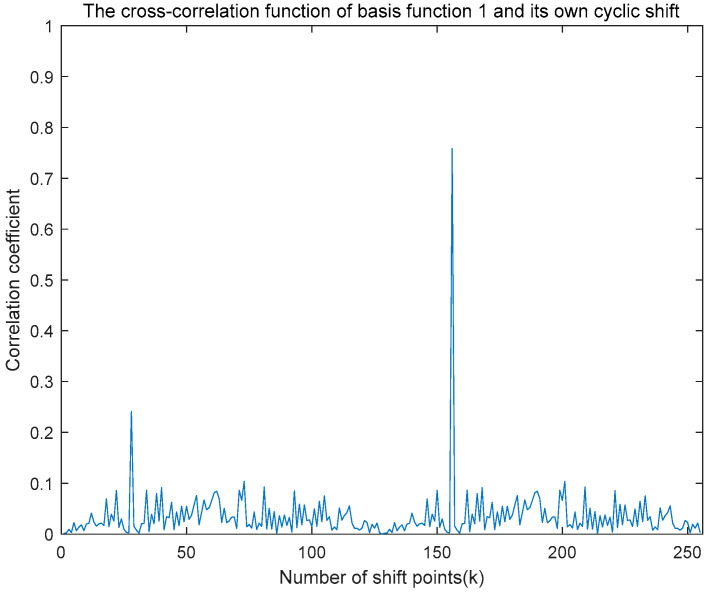
Graph of the cross-correlation function of basis function 1 with its own cyclic shift.

The cross-correlation function between the time-domain basis function and its cyclically shifted version is illustrated in Figure 11, demonstrating the result of a cross-correlation operation with a cyclic shift of 100 bits. This cross-correlation function presents dual peaks, whose positions directly correspond to the number of cyclic shifts. By examining either the first or last half of the unit length of this cross-correlation function, the number of cyclic shifts can be accurately identified based on the relative positions of the correlation peaks. During demodulation at the receiver, the relative positions of these correlation peaks correspond to specific modulation data, thereby facilitating accurate demodulation. Theoretically, a time-domain basis function of length 128 allows for CCSK modulation up to the 128th order. However, practically, considerations such as signal delay and inter-symbol interference, which can cause demodulation errors, generally limit the modulation order to a value lower than 128, such as 64 or 32. This ensures that the correlation peaks can be effectively detected within an acceptable range, thereby reducing the system’s bit error rate.

The correlation characteristics of the time-domain basis functions induced by random-phase mapping are critical. Leveraging these characteristics, correlation-based demodulation can be effectively performed at the receiver, facilitating accurate system demodulation. Moreover, these correlation properties underscore the noise-like nature of the system, significantly reducing detectability and providing a robust foundation for enhancing overall system security.

### 3.2. Error Performance Analysis

The key distinction between the modulation technology of the proposed NRPCS and that of traditional communication systems lies in the modulation method itself. Traditional systems typically employ carrier-based modulation, whereas NRPCS utilizes waveform-based modulation. In NRPCS, the basis function waveform used for modulation is generated through random phase loading in the frequency domain, followed by the elimination of interference frequency components, and subsequently transformed into the time domain. This approach ensures that the modulation retains the essential characteristics of noise-like behavior, along with low probability of interception (LPI) and low probability of detection (LPD) on Reference [21].

To further enhance modulation efficiency and reduce the bit error rate (BER), various schemes have been proposed. These include bipolar modulation, binary Cyclic Code Shift Keying (CCSK), hybrid modulation, and multivariate modulation. The development and integration of these techniques have contributed to the continuous optimization of NRPCS modulation technology, enabling improved performance in covert wireless communication.

In the bipolar modulation scheme of NRPCS, the generated time-domain basis function is directly used to represent bit “1”, while its inverted (negative) version represents bit “0”. This modulation method is analogous to the signal constellation of baseband Binary Phase Shift Keying (BPSK), where “+1” corresponds to bit “1” and “−1” to bit “0”. Consequently, under an additive white Gaussian noise channel, the bit error rate of NRPCS using bipolar modulation can be analyzed by referencing the well-known BER formula for BPSK.

(28) $$P_{b}=Q(\sqrt{\frac{2E_{b}}{N_{0}}})=\frac{1}{2}erfc(\sqrt{\frac{E_{b}}{N_{0}}})$$

where $E_{b}$—symbol energy; $N_{0}$—noise power spectral density;

Q—Complementary cumulative function; the expression is

(29) $$Q(x)=\int_{x}^{+\infty }\frac{1}{\sqrt{2\pi }}e^{-\frac{1}{2}t^{2}}dt$$

erfc(x)—Complementary error function, expressed as

(30) $$erfc(x)=\frac{2}{\sqrt{\pi }}\int_{x}^{+\infty }e^{-t^{2}}dt$$

In binary CCSK modulation, bit “1” is represented by the original time-domain basis function, while bit “0” is represented by a version of the basis function that is cyclically shifted by half of its length. Based on previous analyses and simulation results, it is evident that the time-domain basis function and its cyclically shifted counterpart exhibit approximate orthogonality. As a result, the bit error rate performance of CCSK modulation can be analyzed by referencing the BER expression of Frequency Shift Keying (FSK). Under an additive white Gaussian noise channel, the BER of the NRPCS scheme employing binary CCSK modulation can be expressed as

(31) $$P_{b}=Q(\sqrt{\frac{E_{b}}{N_{0}}})=\frac{1}{2}erfc(\sqrt{\frac{E_{b}}{2N_{0}}})$$

According to the findings presented in Reference [22], the symbol error rate of M-ary CCSK modulation under non-coherent detection can be expressed as follows:

(32) $$P_{s}=1-\int_{-\infty }^{+\infty }e^{-(x+q)}I_{0}(\sqrt{4qx}){\left(1-e^{-x}\right)}^{M-1}dx$$

Assuming that each symbol carries $k=\log_{2}M$ bits and that the signal set consists of orthogonal waveforms with all symbols being equally likely, the theoretical bit error rate of M-ary CCSK modulation under noncoherent detection can be expressed as

(33) $$P_{b}=\frac{2^{k-1}}{2^{k}-1}P_{s}=\frac{2^{\log_{2}M-1}}{2^{\log_{2}M}-1}\left(1-\int_{-\infty }^{+\infty }e^{-(x+q)}I_{0}(\sqrt{4qx}){\left(1-e^{-x}\right)}^{M-1}dx\right)$$

According to relevant research results of Reference [23], the sign error rate of M-input CCSK modulation coherent detection can be expressed as

(34) $$P_{s}=\frac{1}{\sqrt{2\pi }}\int_{-\infty }^{+\infty }\left[1-{\left(\frac{1}{\sqrt{2\pi }}\int_{-\infty }^{y}e^{-\frac{x^{2}}{2}}dx\right)}^{M-1}\right]\;e^{-\frac{1}{2}{\left(y-\sqrt{\frac{2E_{s}}{N_{0}}}\right)}^{2}}dy$$

Similar to the non-coherent demodulation analysis process, the bit error rate of coherent detection of M-input CCSK modulated signals can be expressed as

(35) $$P_{b}=\frac{1}{\sqrt{2\pi }}\frac{2^{\log_{2}M-1}}{2^{\log_{2}M}-1}\int_{-\infty }^{+\infty }\left[1-{\left(\frac{1}{\sqrt{2\pi }}\int_{-\infty }^{y}e^{-\frac{x^{2}}{2}}dx\right)}^{M-1}\right]\;e^{-\frac{1}{2}{\left(y-\sqrt{\frac{2E_{s}}{N_{0}}}\right)}^{2}}dy$$

The time domain basis function and its cyclic shift generated by random phase have the characteristic of Gaussian white noise, and the modulation basis function is approximately orthogonal, so an approximate complete positive intersection can be formed. Therefore, the bit error rate of M-input CCSK modulation using random phase basis function can refer to the upper bound formula of the bit error rate of MFSK modulation mode with each modulation basis function orthogonal on Reference [24], the BER can thus be expressed as

(36) $$P_{b}\left(M\right)\leq \left(M-1\right)Q(\sqrt{\frac{E_{b}}{N_{0}}})$$

(37) $$P_{b}\left(M\right)\leq \frac{1}{2}\left(M-1\right)erfc(\sqrt{\frac{E_{b}}{2N_{0}}})$$

#### 3.2.1. Error Performance Analysis Bipolar Modulation and CCSK Modulation Error Performance Analysis

**Figure 12:**
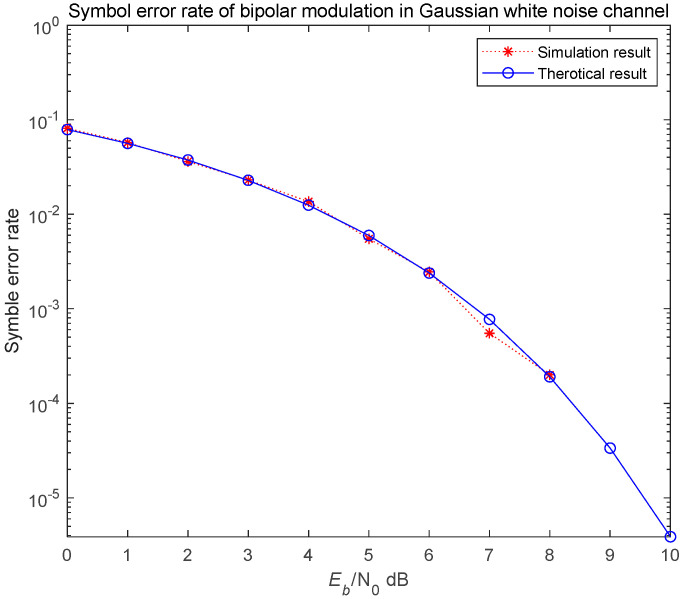
Bit error rate of the system using bipolar modulation.

**Figure 13:**
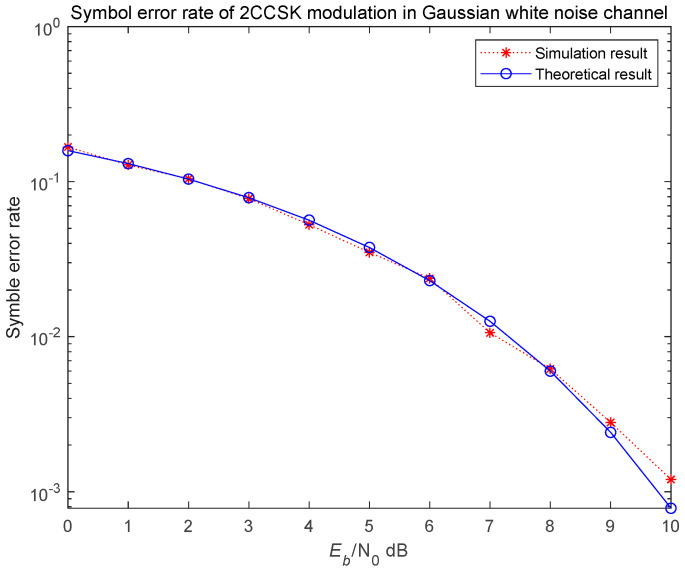
Bit error rate of the system using binary CCSK modulation.

To analyze and simulate the error performance of the NRPCS scheme, we begin by assuming an ideal transmission scenario—free from interference at both the transmitter and receiver ends—and a channel modeled as additive white Gaussian noise. The bit error rate performance of the system is simulated separately for bipolar modulation and binary CCSK modulation using correlation-based demodulation. The length of the time domain basis function generated by random phase was set to N=128, the number of symbols is 5000 and the signal-to-noise ratio varied from 0 to 10 dB. Finally, the bit error rate simulation curve shown in Figure 12 and Figure 13 was obtained.

The simulation results are generally consistent with the theoretical analysis results. According to the bit error rate simulation curves, the NRPCS system employing bipolar modulation demonstrates a significantly better bit error rate performance compared to that using binary CCSK modulation. However, the CCSK modulation scheme offers the advantage of improved spectral efficiency, which cannot be achieved with bipolar modulation.

#### 3.2.2. Error Performance Analysis of Multi-Base CCSK Modulation

**Figure 14:**
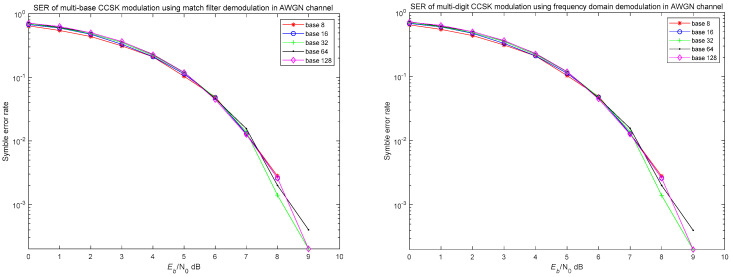
The bit error rate of the system using multi-base CCSK modulation.

The simulation was conducted under ideal conditions, assuming no interference at either the transmitter or receiver, and with the channel modeled as additive white Gaussian noise. At the transmitter, multi-level CCSK modulation was employed, while at the receiver, both frequency-domain demodulation and matched filter demodulation were utilized for comparison. Simulation parameters were set as the length of time domain basis function N=128 generated by random phase, the number of symbols is 5000, the base numbers were set as 8, 16, 32, 64 and 128, respectively, and the signal-to-noise ratio varied from 0 to 10 dB. The simulation results of bit error rate are shown in Figure 14.

The simulation results indicate that the system exhibits relatively poor error performance at low SNR. However, as the SNR increases, the BER improves rapidly. Frequency-domain demodulation and matched filter demodulation yield highly consistent results, which can be attributed to their underlying similarity in principle. This consistency may also be due to the time-domain basis function having a symbol length of 128. When correlation peaks occur at adjacent positions, the system may automatically interpret them as the symbols corresponding to those similar positions. As a result, the BER curves for different modulation orders tend to be relatively concentrated.

A time-domain basis function with a sign length of N(N is a multiple of 2) can theoretically perform CCSK modulation in N-base, with each symbol mapping $k=\log_{2}N$ bit data. All frequencies can be used, the data transmission rate and spectrum utilization efficiency can be improved by $\log_{2}N$ times compared with the direct expansion system. However, in practice, if the symbol length is equal to the modulation order of the multi-base CCSK modulation mode, the system synchronization and timing requirements will be very high, in order to avoid the misjudgment of peak adjustment, so the modulation order M is generally less than N, bringing about a $\log_{2}M$ times increase in modulation efficiency. Therefore, it is very important to set the code length N and modulation order M reasonably if the multi-base CCSK is used in the system design.

### 3.3. Covert Characteristic Analysis

Assuming that nodes A, B, and W operate within the same electromagnetic spectrum environment, there is no mismatch in their spectrum sensing or estimation. It is further assumed that the legitimate transmitter (A) and receiver (B) are capable of generating identical basis function waveforms. Meanwhile, the adversary (W) can detect the subcarriers into which A injects energy by monitoring the electromagnetic spectrum. However, since W does not possess knowledge of the pseudo-random phase generation strategy employed by A and B, it is unable to reconstruct the corresponding time-domain basis function waveforms. As a result, (W) cannot coherently detect or combine the signal energy, thereby failing to demodulate the legitimate signal on Reference [25].

**Figure 15:**
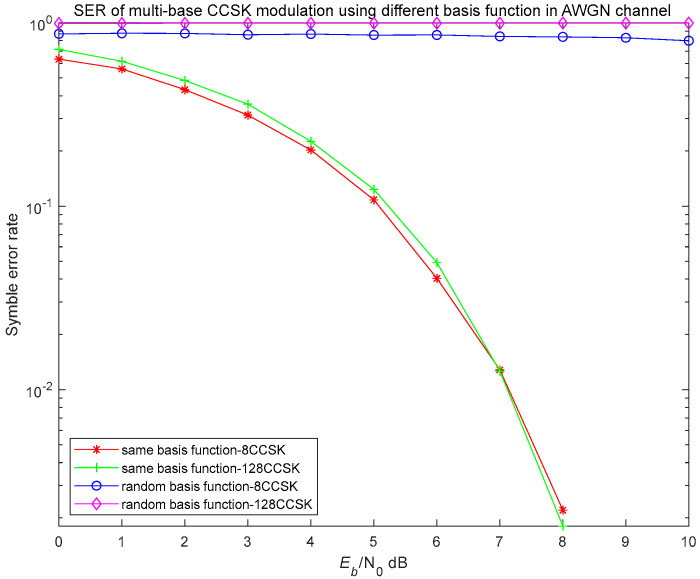
Performance evaluation of covert characteristic.

To further assess the detectability of the signal under conditions where the modulation scheme is unknown, a simulation was conducted. we set that under a Gaussian white noise channel, the number of symbols is 5000, and the SNR varied from 0 to 10 dB. Next, simulation analysis is carried out, as shown in Figure 15. Demodulation is performed using the same time-domain basis function and another different set of time-domain basis functions, respectively.

As shown, when the receiver is unaware of the specific time-domain basis function used by the transmitter, demodulation based on an incorrect random basis function fails to recover any meaningful information, resulting in a bit error rate (BER) of 1. Conversely, when the receiver is synchronized with the transmitter’s pseudo-random sequence and uses the correct basis function, the demodulation performance aligns with theoretical expectations, thereby validating the accuracy and consistency of both the simulation and the analytical model.

Moreover, the BER of the hidden signal decreases as the SNR increases. This is because demodulation errors at the symbol level directly affect the accuracy of the hidden signal recovery. A higher symbol error rate leads to greater interference in the demodulation process, amplifying the BER of the covert signal. Therefore, without knowledge of the exact modulation method or basis function generation strategy, an adversary cannot successfully intercept or decode the transmitted signal. This effectively prevents unauthorized access and malicious activities, thereby ensuring the covert nature of the communication and significantly enhancing the security and reliability of signal transmission.

## 4. Conclusions

This paper proposes a novel stealth communication scheme based on multi-carrier noise-like random phase modulation. The time-domain basis function employed in this approach exhibits excellent autocorrelation and cross-correlation characteristics. Specifically, the autocorrelation function presents a sharp and narrow peak, indicating strong temporal coherence and high signal concentration. Meanwhile, the cross-correlation function approaches zero, implying minimal inter-signal interference. These favorable correlation properties enhance the system’s anti-interference capabilities, reduce inter-symbol interference, improve synchronization accuracy, and increase spectral efficiency, thereby supporting efficient modulation, demodulation, and multi-user access.

Furthermore, the power spectral density of the noise-like random phase waveform closely approximates that of Gaussian white noise, exhibiting a noise-like, featureless profile. Similarly, the time-domain waveform also mimics random noise, enabling effective concealment within Gaussian noise environments and meeting the requirements for low probability of detection (LPD) and low probability of interception (LPI). The integration of advanced multi-level CCSK modulation further enhances the modulation efficiency of the system.

In addition to improving anti-interception performance, this study also addresses the challenge of Peak-to-Average Power Ratio (PAPR), which is inherent in noise-based modulation schemes. The random phase characteristics may result in large instantaneous power fluctuations, leading to elevated PAPR levels. High PAPR can adversely affect power efficiency, amplifier linearity, bit error rate, and overall transmission quality. To mitigate this, techniques such as signal mapping, selective mapping, and signal clipping are introduced. These methods effectively reduce PAPR, optimize system performance, and improve power efficiency. Proper PAPR control contributes to reducing power consumption, minimizing bit error rate, and further enhancing the system’s resistance to interference. These improvements are of significant importance for the overall performance and reliability of modern communication systems, particularly in stealth and covert communication scenarios. PAPR control will be a key focus in future research.

In the context of multi-carrier noise-like random phase modulation systems, the proposed method ensures that, without prior knowledge of the specific modulation scheme and the generated time-domain basis function used by the transmitter, the receiver cannot recover the transmitted information. The resulting stealth signal, characterized by low energy and Gaussian-like properties, provides strong resistance against detection and interception, thereby enabling highly covert communications. Compared to traditional covert communication techniques such as spread spectrum modulation, the waveform of the proposed scheme more closely resembles Gaussian white noise in both the frequency and time domains, making it significantly more difficult to detect in noisy environments. This allows the system to maintain strong concealment capabilities even under low signal-to-noise ratio (SNR) conditions.

Overall, this work presents a promising direction for the development of advanced covert communication technologies, with substantial potential for application in secure communication systems and other sensitive domains.

## Figures and Tables

**Table 1:** CCSK modulation mapping method with sequence length N=32 modulation order M=32.

Data to Be Called	CCSK Element
00000	*b*_0_ *b*_1_ *b*_2_ … *b*_30_ *b*_31_
00001	*b*_1_ *b*_2_ *b*_3_ … *b*_31_ *b*_0_
00010	*b*_2_ *b*_3_ *b*_4_ … *b*_0_ *b*_1_
…	…
11111	*b*_31_ *b*_0_ *b*_1_ … *b*_29_ *b*_30_

**Table 2:** CCSK modulation mapping method with sequence length N=128 modulation order M=32.

Data to Be Called	CCSK Element
00000	*b*_0_ *b*_1_ *b*_2_ *b*_3_ *b*_4_ *b*_5_ *b*_6_ *b*_7_ … *b*_124_ *b*_125_ *b*_126_ *b*_127_
00001	*b*_4_ *b*_5_ *b*_6_ *b*_7_ *b*_8_ *b*_9_ *b*_10_ *b*_11_ … *b*_0_ *b*_1_ *b*_2_ *b*_3_
00010	*b*_8_ *b*_9_ *b*_10_ *b*_11_ *b*_12_ *b*_13_ *b*_14_ *b*_15_ … *b*_4_ *b*_5_ *b*_6_ *b*_7_
…	…
11111	*b*_124_ *b*_125_ *b*_126_ *b*_127_ *b*_0_ *b*_1_ *b*_2_ *b*_3_ … *b*_120_ *b*_121_ *b*_122_ *b*_123_

## Data Availability

The original contributions presented in this study are included in the article. Further inquiries can be directed to the corresponding author.

## Abbreviations

The following abbreviations are used in this manuscript:

LPD	Low Probability of Detection
LPI	Low Probability of Interception
NRPCS	Noise-like Random Phase Communication System
CCSK	Cyclic Code Shift Keying
WFRFT	Weighted-type Fractional Fourier Transform
MPWFRFT	Multi-parameter Weighted-type Fractional Fourier Transform
PAPR	Peak to Average Power Ratio
FMW	Fundamental Modulation Waveform
OFDM	Orthogonal Frequency Division Multiplexing
PSD	Power Spectrum Density
LFSR	Linear Feedback Shift Register
